# Evaluating the role of montelukast on doxorubicin-induced cardiotoxicity in breast cancer patients

**DOI:** 10.1007/s00520-025-09947-z

**Published:** 2025-10-01

**Authors:** Naglaa F. Gomaa, Rehab H. Werida, Ahmed G. EL-Gowily, Noha A. El-Bassiouny

**Affiliations:** 1 Department of Clinical Pharmacy and Pharmacy Practice, Faculty of Pharmacy, Damanhour University, Damanhour, Egypt; 2 Department of Clinical Oncology, Faculty of Medicine, Alexandria University, Alexandria, Egypt

**Keywords:** Montelukast, Cardiotoxicity, Breast cancer, Anthracycline

## Abstract

**Purpose:**

Doxorubicin (DOX), a prominent anthracycline, is used to treat malignancies, but its cardiotoxicity restricts its therapeutic application. This study examined the potential protective effects of montelukast (ML), an anti-asthmatic drug with anti-inflammatory characteristics, against doxorubicin-induced cardiotoxicity (DIC) in breast cancer (BC) patients.

**Method:**

A prospective, randomized, controlled clinical study including fifty individuals with a confirmed diagnosis of BC, individuals scheduled to receive DOX 60 mg/m^2^ in conjunction with Cyclophosphamide 600 mg/m^2^ (AC) for four courses at 21-day intervals. Both the control group and the ML group were randomly selected from the patient pool.

**Results:**

After treatment, a significant reduction in N-terminal Pro Brain Natriuretic Peptide (NT pro-BNP) levels was observed in the ML group compared to the control group (1756.0 [1054.0–2334.0] vs. 3788.0 [2226.0–4401.1] pg/mL, p < 0.001). Nuclear factor-kappa B (NF-κB) levels also decreased significantly in the ML group (2.23 [1.18–3.05] vs. 3.11 [2.39–3.25] pg/mL, p = 0.009). The median percentage reduction in Soluble suppression of tumorigenicity 2 (sST2) levels was more pronounced in the ML group (20.93 ± 5.45 ng/mL) than in the control group (24.16 ± 5.14 ng/mL, p = 0.036). Additionally, a strong positive correlation between NT pro-BNP and NF-κB levels was observed post-treatment (rs = 0.644, p < 0.001), supporting ML’s potential anti-inflammatory and cardioprotective effects.

**Conclusion:**

The incorporation of ML into AC led to a substantial decrease in cardiac biomarkers confirming the feasibility of incorporating ML in individuals with breast cancer as an auxiliary treatment to prevent DOX-induced cardiotoxicity.

**Trial registration** ClinicalTrials.gov: NCT05959889.

## Introduction

Breast cancer (BC) continues to be the largest cause of cancer-related death despite improvements in its identification and treatment, especially for women of color [1]. According to the GLOBOCAN 2022 report, BC was responsible for 6.9% of cancer-related deaths worldwide and 11.6% of all cancer diagnoses [2]. Although therapies can increase survival rates, they frequently result in cardiac issues that have a negative impact on general health outcomes [3]. Environmental factors, lifestyle decisions, genetic predispositions, and demographics are some of the risk factors affecting BC [4, 5].

Anthracyclines play a crucial role in BC treatment due to their effectiveness in improving patient survival rates [6]. Doxorubicin (DOX), a widely used anthracycline, is one of the most effective and cost-efficient options for treating various solid tumors and blood cancers [7]. However, the administration of this substance is linked to recognized cardiotoxic effects, potentially leading to severe consequences including left ventricular dysfunction and heart failure [8, 9]. Cardiotoxicity from anthracyclines is still a serious clinical issue, with the risk primarily associated with cumulative dosage [10]. Pre-existing cardiovascular issues, advanced age, and prior thoracic radiation are further risk factors [11]. Improvements in biomarkers and cardiac imaging have enhanced its early detection [12, 13].

The cardiovascular risks of anthracyclines can be divided into three categories: acute, early-onset chronic, and late-onset chronic [14, 15]. Acute cardiotoxicity may be present in some individuals shortly after treatment commencement; Chronic cardiotoxicity may manifest in two ways: early-onset, which occurs during the initial year after therapy, and late-onset, which can occur years later [16]. Numerous mechanisms contribute to doxorubicin-induced cardiotoxicity (DIC), such as inflammation, apoptosis, and oxidative stress [17, 18]. Reactive oxygen species (ROS) significantly contribute to heart dysfunction and mitochondrial impairment, often leading to the cessation of medication [19].

Montelukast (ML), a recognized cysteinyl leukotriene (CysLT) antagonist mostly used for the treatment of asthma and allergic rhinitis, has antioxidant and anti-inflammatory characteristics [20]. Studies indicated that ML has protective effects across various inflammatory models, including colitis and kidney toxicity [21, 22]. There is growing interest in repurposing CysLT receptor antagonists (CysLTRA) like ML for cardiovascular diseases (CVDs) [23]. CysLTs are implicated in several conditions such as myocardial infarction (MI) and atherosclerosis [24–26]. The anti-inflammatory properties of ML have led researchers to believe that it may enhance heart function after myocardial damage and decrease myocardial harm from different stressors [27–29].

Preclinical research demonstrated ML's ability to treat cardiovascular problems and demonstrated its wider anti-inflammatory advantages beyond pulmonary disorders [30]. For example, ML has demonstrated the capacity to decrease monocyte adhesion to endothelial cells caused by oxidized low-density lipoprotein (ox-LDL) and to slow the progression of atherosclerosis by inhibiting specific inflammatory mediators [31]. ML decreased ROS levels in cardiac tissues in studies including DOX administration, indicating that it might be a useful cardioprotective agent during chemotherapy [32]. Additionally, ML has been effective in mitigating DOX-induced testicular damage by lowering oxidative stress and inflammation [33].

Studies conducted in clinical settings have shown that BC patients who express high levels of CysLT receptor 1 (CysLT1R) tend to have poorer outcomes [34]. Furthermore, research has indicated that CysLT1RA may help reduce cardiovascular events, including ischemic strokes [35]. ML usage has been associated in observational studies with a lower incidence of major ischemic cardiovascular events in asthmatic patients [36, 37].

To identify and treat cardiovascular diseases, particularly in patients receiving DOX, where the possibility of cardiotoxicity is a significant issue, cardiac function assessment is crucial. Early detection and management of chemotherapy-induced cardiac impairment is facilitated by various cardiac biomarkers such as N-terminal pro B-type Natriuretic Peptide (NT-proBNP), which is a crucial indicator of cardiac stress, and elevated levels of this biomarker have been connected to the cardiotoxic effects of chemotherapeutic agents like anthracyclines [38]. Heart hypertrophy, heart failure, and chemotherapy-induced cardiotoxicity are all influenced by Nuclear Factor kappa (NF-κB), which is crucial for inflammation and cell viability [39]. Furthermore, elevated levels of Soluble Suppression of Tumorigenicity 2 (sST2), a biomarker linked to cardiac remodeling, inflammation, and fibrosis, correlate with harmful cardiac remodeling and fibrosis. This might indicate the cardiotoxic effects of treatments and serve as a gauge of the prognosis for heart failure [40]. Studies on the effects of ML on DIC are currently limited, particularly in BC communities. To better understand cardiotoxicity-related heart injury and create a successful management plan, these three biomarkers were assessed in this study. By investigating the potential preventive advantages of ML against DIC in BC patients for the first time, this study aims to address this significant gap. The primary objective was to measure levels of biomarkers such as NT-proBNP, NF-κB and sST2 to assess the effect of ML on DIC in BC patients. The secondary objective focused on evaluating the risk profile and side effects associated with ML usage in these people.

## Patients and methods

### Study design

Participants were recruited from Damanhour Oncology Center in Egypt for this randomized, controlled clinical investigation, which was intended to be conducted in parallel groups. The study protocol was authorized by the Damanhour University Research Ethical Committee (Approval number: 123PP64) and the Research Ethics Committee of the Ministry of Health (Approval Number: 2–2024/12). Additionally, it was registered at ClinicalTrials.gov“https://www.clinicaltrials.gov/study/NCT05959889?cond=breast%20cancer&term=Montelukast&rank=1” with Clinical Trial Registry: NCT05959889 (Study Registration Dates 2023–07-16). All study protocols followed the Declaration of Helsinki, and patients were required to sign informed permission forms before participating in the study.

### Study participants

119 individuals were eventually chosen during the study's eligibility review phase, which ran from March 2023 to December 2024. Ultimately, 50 patients, 25 in the control group and 25 in the ML group, were enrolled to assess the study's main and secondary outcomes. The research included adult females (ranging in age from 18 to 70 years) who had a BC diagnosis that was verified by a biopsy, according to the TNM staging criteria by the American Joint Committee on Cancer [41] and met the following criteria: scheduled to be treated with AC regimen, their ECOG performance status was between 0 and 2, and had adequate hematological, hepatological, and renal functions. Individuals with significant liver problems, recent heart attacks, cardiac conditions (including angina pectoris, uncontrolled hypertension, arrhythmia, and left ventricular ejection fraction < 50%), or a history of treatment with specific anticancer medications were excluded from the study. Patients who had signs of metastases during the initial evaluation were also ineligible, as were women who were pregnant or breastfeeding.

### Randomization and study interventions

Eligible patients were assigned in a 1:1 ratio to the Control or ML groups using block randomization. Four days before AC therapy began, each patient was assigned to one of the two groups. The control group received 4 cycles of the AC regimen which consisted of an intravenous (IV) infusion of DOX (dose/cycle = 60 mg/m^2^) administered as a slow IV push over 5–10 min, followed by an infusion of cyclophosphamide (dose/cycle = 600 mg/m^2^) over 30–60 min, with a 21-day interval [42]. The ML group received the same chemotherapy protocol in addition to 10 mg of ML (Asmakast®) once daily at bedtime. Direct, in-person patient interviews, reviews of medical records, and self-reports were used to gather data. Sociodemographic characteristics that were analyzed included age, gender, marital status, place of residence, and history of cancer. Cancer grade, BC type, chemotherapy (adjuvant or neoadjuvant), cycle interval, comorbidities, and ECOG performance status were among the clinical data [43].

### Blood sampling and biochemical analyses

Antecubital venipuncture was used to obtain approximately 5 mL of venous blood from each participant at two different times: approximately one hour prior to the first chemotherapy cycle (Baseline) and one hour after the final AC dosage (After). Blood samples were collected in EDTA test tubes, centrifuged for 10 min at 4,500 × g, and then stored at −80 °C. We used enzyme-linked immunosorbent assay (ELISA) kits that were purchased from DL develop (Wuxi Donglin Sci & Tech Development Co., Ltd., China) and Sun Red Biotechnology Co., Ltd., China, to measure the plasma levels of NT-proBNP, NF-κB, and sST2. The respective catalog numbers for the kits were DL-NT-ProBNP-Hu, DL-NFkB-Hu, and 201–12-5940. Every assay was carried out in compliance with the manufacturer's instructions.

### Patient assessment and follow-up schedule

ML treatment began with the patients' first AC dosage and continued concurrently for four cycles of AC doses, ending after their fourth dosage. The drug was provided to the patients every 21 days, in accordance with their revised AC dosage. Adherence was tracked via drug refill rates, bi-weekly phone calls, and direct meetings every 21 days at the time of their prescribed AC doses and to report any adverse effects. To assess adherence, pill counts were used. Patients were eliminated from the trial after being judged non-compliant if they received less than 90% of their medication. Notably, no participants were excluded due to non-compliance, and the medication adherence rate was 100%, with all participants following their prescribed regimen precisely.

### Sample size calculations

The sample size was estimated based on a previous study [44]. G power software was used to perform the power analysis by T tests; the criteria for significance was set at 0.05 (type I error) and the total power was 90%; the effect size was set at 0.95 based on the difference in NT-proBNP between the ML and control groups; the total sample size required was 50 and the allocation ratio was 1 so, the total sample size was divided into 25 in each group.

### Statistical analysis

The results were analyzed with the assistance of IBM SPSS version 20.0. The standard deviation, median, and interquartile range were used to express the quantitative data. However, qualitative data was represented by percentages and numbers. Statistical significance was set at a 5% significance level. Group differences were evaluated using the following statistical tests: the Student t-test for normally distributed quantitative variables, Fisher's Exact Test Correction for chi-square tests when more than 20% of cells had expected counts that were less than five, the Mann–Whitney for non-normally distributed quantitative variables, and the Wilcoxon signed-rank ranks for categorical variables.

## Results

**Fig. 1:**
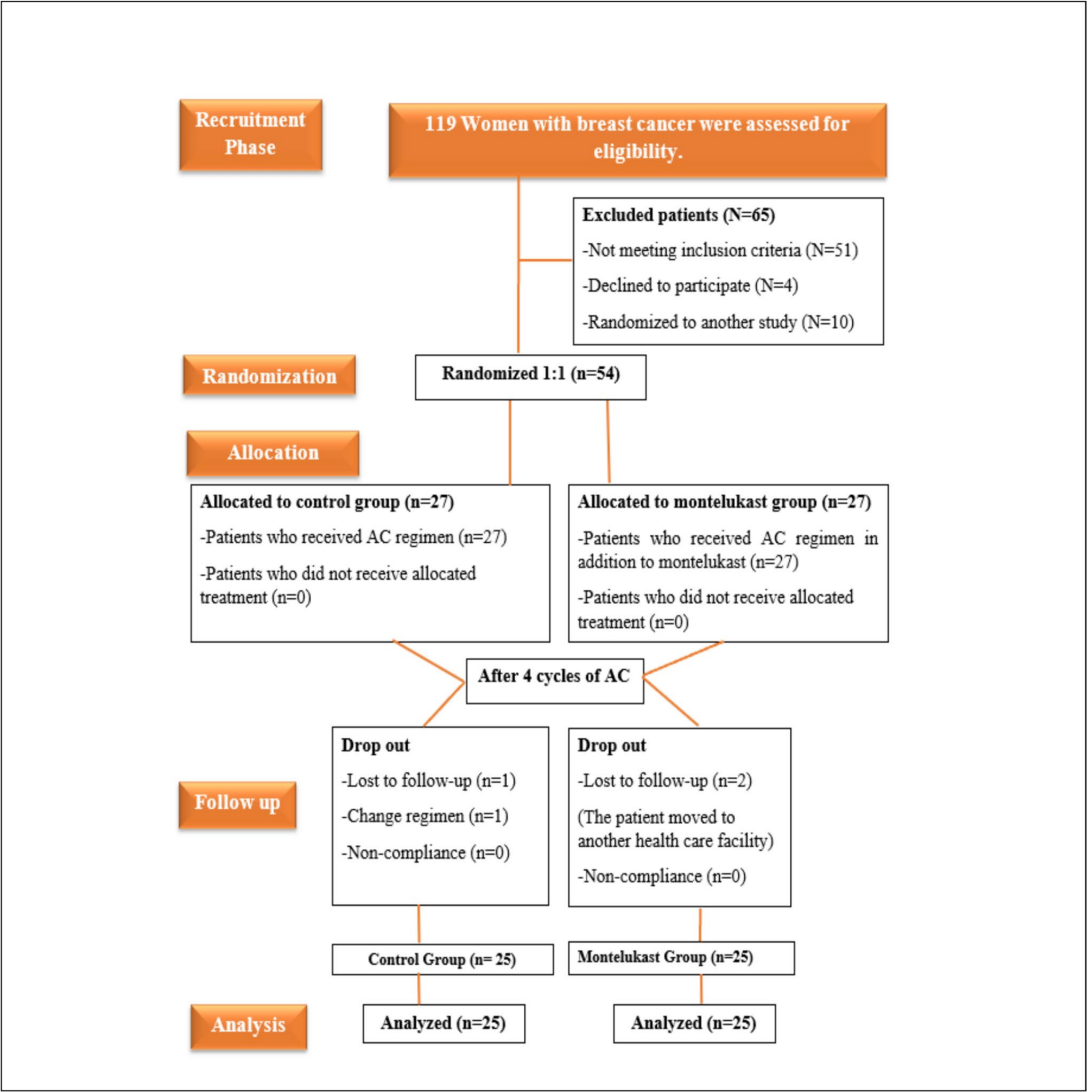
The Consolidated Standards of Reporting Trials **(CONSORT)** diagram

Out of the 119 participants screened, 4 declined to participate, 10 were enrolled in a different trial, and 51 were not eligible to participate. Ultimately, 54 patients who satisfied the inclusion requirements were enrolled in the study. The individuals were then randomized to either the Control or ML groups. During the follow-up period, patients were excluded from the trial due to three instances of lost follow-up, one instance of regimen change, and zero exclusions related to non-compliance. The **CONSORT** diagram, which summarizes patient enrollment, randomization, and follow-up throughout the trial, is shown in Fig. 1.

### Characteristics of the baseline population: demographic and clinical

Age, weight, height, and BMI were among the demographic variables that showed no statistically significant differences between the study groups. Furthermore, there were no significant differences in the type of therapy (adjuvant/neoadjuvant), family history, contraception method, breastfeeding status, or menopausal status. Likewise, as Table 1 illustrates, there were statistically insignificant differences between the two groups' baseline clinical and tumor characteristics.

**Table 1:** Demographic and clinical characteristics in control and ML groups

	Control group(*n* = 25)	ML group (*n* = 25)	*p*
Age (years)	47.36 ± 14.56	42.56 ± 9.95	^t^p = 0.181
Weight (kg)	79.48 ± 12.78	74.56 ± 13.50	^t^p = 0.192
Height (m)	1.66 ± 0.05	1.63 ± 0.06	^t^p = 0.080
BMI (Kg/m^2^)	28.92 ± 4.17	28.03 ± 4.62	^t^p = 0.479
Menopausal			
Premenopausal	15 (60.0%)	20 (80.0%)	^a^p = 0.123
Postmenopausal	10 (40.0%)	5 (20.0%)	
Adjuvant/Neoadjuvant			
Adjuvant	19 (76.0%)	17 (68.0%)	^a^p = 0.529
Neoadjuvant	6 (24.0%)	8 (32.0%)	
Family history	4 (16.0%)	9 (36.0%)	^a^p = 0.107
Contraception method			
Nothing	3 (12.0%)	3 (12.0%)	^FE^p = 1.000
Hormonal	7 (28.0%)	7 (28.0%)	
Non-Hormonal	12 (48.0%)	12 (48.0%)	
Both	3 (12.0%)	3 (12.0%)	
Breastfeeding Status	23 (92.0%)	23 (92.0%)	^FE^p = 1.000
Side			
Right	10 (40.0%)	11 (44.0%)	^FE^p = 1.000
Left	14 (56.0%)	14 (56.0%)	
Both	1 (4.0%)	0 (0.0%)	
Receptor status			
ER +	15 (60.0%)	19 (76.0%)	^a^p = 0.225
PR +	15 (60.0%)	19 (76.0%)	^a^p = 0.225
HER2 +	8 (32.0%)	7 (28.0%)	^a^p = 0.758
Concomitant diseases			
Diabetes	0 (0.0%)	3 (12.0%)	^FE^p = 0.235
HTN	4 (16.0%)	3 (12.0%)	^FE^p = 1.000
Obesity	2 (8.0%)	1 (4.0%)	^FE^p = 1.000
Grade			
II	18 (72.0%)	14 (56.0%)	^a^p = 0.239
III	7 (28.0%)	11 (44.0%)	
Clinical prognostic stage			
IA	2 (8.0%)	1 (4.0%)	^FE^p = 0.900
IIA	6 (24.0%)	4 (16.0%)	
IIA or IIB	0 (0.0%)	1 (4.0%)	
IIB Right/IIA Left	1 (4.0%)	0 (0.0%)	
IIB	8 (32.0%)	9 (36.0%)	
IIIA	4 (16.0%)	6 (24.0%)	
IIIB	4 (16.0%)	3 (12.0%)	
IIIC	0 (0.0%)	1 (4.0%)	
Ki-67 (%)	30.0 (20.0 - 40.0)	30.0 (25.0 - 40.0)	^U^p = 0.633
SBP (mmHg)	119.6 ± 2.0	120.0 ± 0.0	^t^p = 0.327
DBP (mmHg)	79.60 ± 2.0	80.0 ± 0.0	^t^p = 0.327
LVEF (Baseline)	65.36 ± 3.25	67.12 ± 3.18	^t^p = 0.059

*BMI* Body Mass Index, *HTN* Hypertension, *SBP* Systolic Blood Pressure, *DBP* Diastolic Blood Pressure, *LVEF* Left Ventricular Ejection Fraction. Qualitative data were described using Number and Percent, and normally, quantitative data were expressed in Mean ± SD. While non-normally distributed data was expressed in Median (IQR), *IQR* Interquartile range. *SD* Standard deviation, *t* Student t-test, *U* Mann–Whitney test, *a* Chi-square test, *FE* Fisher’s Exact test, *p* p-value for comparing between the studied groups, * Statistically significant at p ≤ 0.05

### Effect of ML on plasma levels of NT-proBNP, NF-κB, and sST2 biomarkers

**Fig. 2:**
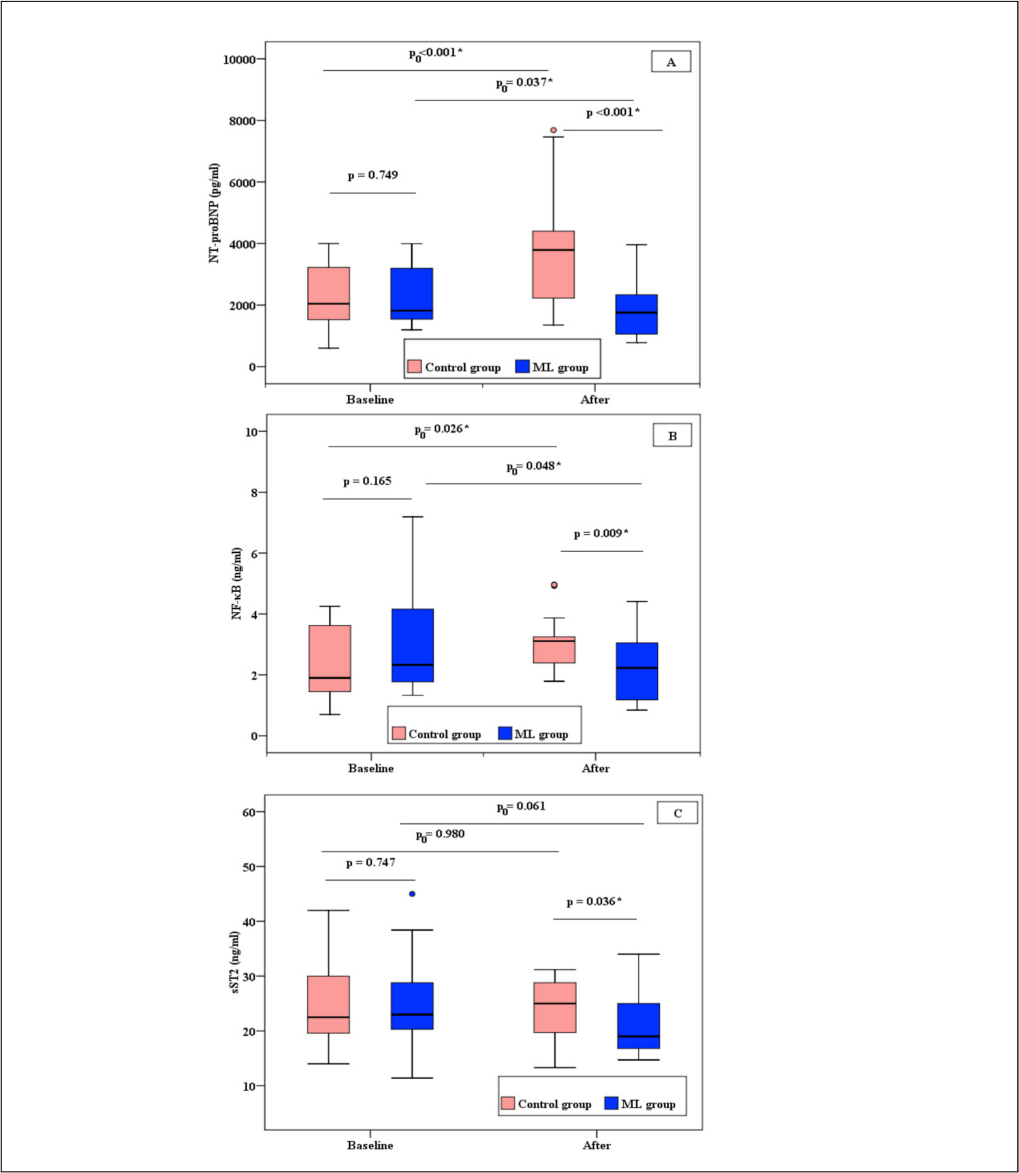
Comparison between the two groups according to **A)** NT-proBNP**, B)** NF-κB and **C)** sST2. **p:** p-value for comparing between the studied groups in each period. **p**_**0**_**:** p-value for comparing between Baseline and After treatment in each group. *: Statistically significant at p ≤ 0.05

The three biomarker levels changed significantly when ML was added to AC therapy, and there were noticeable variations between the ML and Control groups. Regarding the NT-proBNP biomarker analysis, Fig. 2A revealed that the most striking finding was the divergent response in NT-proBNP levels; At baseline, there was an insignificant difference in NT-proBNP levels between the two groups (p = 0.749). After treatment, the control group exhibited a significant increase in median values (2040.9 vs 3788.0 pg/mL, p < 0.001), while the ML group showed a significant reduction (1817.2 vs 1756.0 pg/mL, p = 0.037). The between-group comparison by the end of treatment revealed a statistically significant difference, with the ML group achieving much lower levels compared to the control group (1756.0 [1054.0–2334.0] vs. 3788.0 [2226.0–4401.1] pg/mL, p < 0.001).

Examining the inflammatory marker NF-κB revealed that the control group had significantly higher levels of the marker, with a median of 3.11 pg/mL (p = 0.026). However, as illustrated in Fig. 2B, the ML group showed a significant reduction to 2.23 pg/mL (p = 0.048). Moreover, The ML group showed a significantly lower NF-κB levels after treatment compared to the control group (2.23 [1.18–3.05] vs. 3.11 [2.39–3.25] pg/mL, p = 0.009).

Regarding the sST2 biomarker, the control and ML groups' baseline sST2 levels did not differ significantly (p = 0.747). Likewise, there was no significant change in the control group's sST2 levels before and after therapy (p = 0.980). Even though the ML group's sST2 levels decreased (24.91 ± 7.91 vs. 20.93 ± 5.45), the difference was not statistically significant (p = 0.061). However, following treatment, the ML group's sST2 levels (20.93 ± 5.45 ng/mL) demonstrated a significant decrease (p = 0.036) in comparison to its corresponding control group (24.16 ± 5.14 ng/mL), as illustrated in Fig. 2C.

### The correlations between NT-proBNP, NF-κB, and sST2 levels

**Fig. 3:**
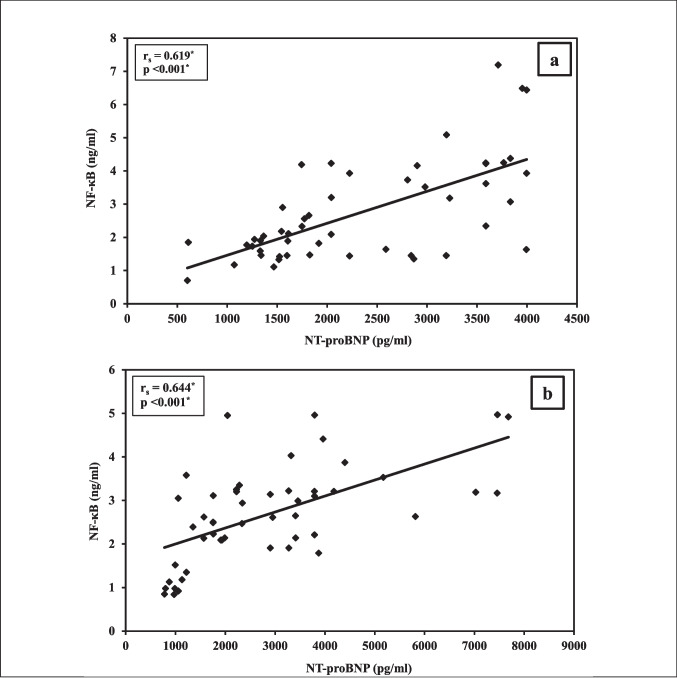
Correlation between NT-proBNP and NF-κB at **a**) baseline and **b**) after treatment in study participants (*n* = 50)

A non-significant correlation was demonstrated between sST2 and both NT-proBNP and NF-κB either before or after the treatment, Table 2 on the other hand, a significant positive correlation was revealed between NT-proBNP and NF-κB before (rs = 0.619, p < 0.001) and after therapy (rs = 0.644, p < 0.001; Fig. 3a and 3b).

**Table 2:** Correlation between NT-proBNP, NF-κB, and sST2 biomarkers (n = 50)

	Baseline		After	
	r_s_	P	r_s_	*P*
NT-proBNP vs. NF-κB	0.619^*^	< 0.001^*^	0.644^*^	< 0.001^*^
NT-proBNP vs. sST2	−0.095	0.511	0.138	0.338
NF-κB vs. sST2	−0.054	0.708	0.104	0.471

*r*_*s*_ Spearman coefficient. * Statistically significant at p ≤ 0.05

### The effect of ML on the adverse events of both studied groups

The safety results showed that, in terms of adverse events, the ML group and the control group exhibited similar profiles. Non-significant differences were found in common symptoms such as fatigue, dyspepsia, headache, dizziness, constipation, diarrhea, abdominal spasms, nausea, vomiting, oral mucositis, hypotension, conjunctivitis, chest discomfort, and taste alteration (p > 0.05). However, the ML group experienced less arm lymphedema (24% vs. 60%) (p = 0.010). Furthermore, hair loss was uniformly incomplete across participants, and no cases of depression were reported, as observed in Table 3.

**Table 3:** The effect of ML on the adverse events of the two studied groups

Side Effects	Control group (*n* = 25)	ML group (*n* = 25)	*P*
Fatigue^b^	22 (88.0%)	20 (80.0%)	0.702
Dyspepsia^b^	3 (12.0%)	3 (12.0%)	1.000
Dizziness^b^	1 (4.0%)	1 (4.0%)	1.000
Constipation^a^	17 (68.0%)	12 (48.0%)	0.152
Diarrhea^a^	14 (56.0%)	12 (48.0%)	0.571
Abdominal spasm^a^	18 (72.0%)	16 (64.0%)	0.544
Nausea^b^	24 (96.0%)	22 (88.0%)	0.609
Vomiting^a^	20 (80.0%)	18 (72.0%)	0.508
Oral Mucositis^a^	10 (40.0%)	9 (36.0%)	0.771
Hypotension^a^	15 (60.0%)	13 (52.0%)	0.569
Blue Fingers^b^	2 (8.0%)	1 (4.0%)	1.000
Conjunctivitis^a^	16 (64.0%)	18 (72.0%)	0.544
Chest Pain^b^	4 (16.0%)	3 (12.0%)	1.000
Headache^a^	17 (68.0%)	16 (64.0%)	0.765
Taste alteration^a^	10 (40.0%)	8 (32.0%)	0.556
Lymphedema of the arm^a^	15 (60.0%)	6 (24.0%)	0.010^*^
Hair loss	25 (100.0%)	25 (100.0%)	–
Depression	0 (0.0%)	0 (0.0%)	–

Qualitative data were described using Number and Percent, and were compared using Chi-square test or Fisher’s Exact test

*a* Chi-square test. *b* Fisher’s Exact test. * Statistically significant at p ≤ 0.05

### The effect of ML on different biological parameters

Hematological and biochemical analyses revealed notable alterations within each group, but no discernible differences between the ML and control groups were observed throughout the cycles. Regarding the hematological parameters, hemoglobin, RBCs, and hematocrit all exhibited a significant intra-group decrease (p < 0.001), whereas MCV, MCH, and MCHC in both the control and ML groups showed non-significant changes (p > 0.05). Both the control and ML groups experienced a significant increase in RDW/CV with time (p = 0.002 and p < 0.001, respectively). Regarding WBC count, there were no significant differences between the control and ML groups; nevertheless, both groups showed a significant decrease over time (p < 0.001 and p = 0.003). Compared to the control group, the ML group's platelet counts changed significantly (p < 0.001). Neutrophil and lymphocyte count showed significant within-group changes without inter-group differences. In terms of the liver function tests, AST demonstrated a significant decrease in cycle 3 when compared to its comparable control (p = 0.005); however, ALT did not change significantly between the control and ML groups across the cycles. Both ALT and AST levels within the ML group showed intragroup variation across the cycles, with a significant elevation observed (p = 0.009 for ALT and < 0.001 for AST), but the control group showed no significant changes. Kidney functions including serum creatinine and urea levels showed no significant differences between or within groups across the cycles (p > 0.05). Also, the fasting plasma glucose levels showed non-significant differences either within the same group or between the control or ML groups, Table 4.

**Table 4:** Comparison between control and ML groups according to different biological parameters in each cycle

		Cycle 1	Cycle 2	Cycle 3	Cycle 4	p_0_
Red Blood Cell (RBC) Parameters						
Hemoglobin (g/dl)	Control group	12.79 ± 1.39	12.36 ± 1.08	12.26 ± 0.89	11.84 ± 1.0	^F^p < 0.001^*^
	ML group	12.70 ± 1.12	11.93 ± 1.06	11.96 ± 1.04	11.85 ± 0.95	^F^p < 0.001^*^
	^t^p	0.798	0.159	0.274	0.977	
RBCs (10^6^/μl)	Control group	4.84 ± 0.67	4.60 ± 0.53	4.56 ± 0.60	4.38 ± 0.58	^F^p < 0.001^*^
	ML group	4.81 ± 0.63	4.45 ± 0.40	4.45 ± 0.48	4.32 ± 0.54	^F^p < 0.001^*^
	^t^p	0.880	0.255	0.471	0.688	
Hematocrit (%)	Control group	38.83 ± 4.22	37.82 ± 2.94	37.26 ± 3.53	35.92 ± 3.24	^F^p = 0.001^*^
	ML group	38.60 ± 3.22	36.60 ± 2.82	36.20 ± 3.12	36.06 ± 3.08	^F^p < 0.001^*^
	^t^p	0.828	0.142	0.268	0.869	
MCV (FL)	Control group	81.03 ± 6.41	82.14 ± 6.23	82.31 ± 7.19	82.84 ± 7.31	^F^p = 0.418
	ML group	81.11 ± 5.72	82.60 ± 6.14	82.11 ± 6.64	84.20 ± 6.67	^F^p = 0.063
	^t^p	0.963	0.794	0.921	0.496	
MCH (pg)	Control group	26.73 ± 2.17	26.93 ± 1.79	27.30 ± 2.36	27.31 ± 2.68	^F^p = 0.096
	ML group	27.0 ± 2.54	27.07 ± 1.81	27.07 ± 1.82	27.66 ± 1.77	^F^p = 0.081
	^t^p	0.686	0.784	0.698	0.591	
MCHC (g/dL)	Control group	32.93 ± 1.39	32.67 ± 1.04	33.0 ± 1.79	33.05 ± 1.96	^F^p = 0.813
	ML group	33.29 ± 2.61	32.63 ± 1.87	33.02 ± 1.66	32.41 ± 2.28	^F^p = 0.427
	^t^p	0.546	0.926	0.961	0.296	
RDW/CV (%)	Control group	14.92 ± 1.34	14.37 ± 1.67	15.22 ± 1.86	15.87 ± 2.03	^F^p = 0.002^*^
	ML group	14.49 ± 1.52	14.45 ± 1.83	15.17 ± 2.33	15.78 ± 2.42	^F^p < 0.001^*^
	^t^p	0.301	0.873	0.936	0.885	
Platelet Parameter						
PLT (× 10^3^/μl)	Control group	321.4 ± 180.41	397.0 ± 150.73	375.9 ± 109.80	364.3 ± 104.15	^F^p = 0.118
	ML group	289.8 ± 71.51	398.0 ± 123.96	350.7 ± 80.54	353.3 ± 95.35	^F^p < 0.001^*^
	^t^p	0.420	0.980	0.359	0.698	
White Blood Cell (WBC) Parameters						
WBCs (× 10^3^/μl)	Control group	8.0 ± 3.24	7.33 ± 3.28	6.10 ± 1.69	5.87 ± 1.46	^F^p < 0.001^*^
	ML group	7.01 ± 2.41	5.81 ± 2.50	5.26 ± 1.35	5.45 ± 1.91	^F^p = 0.003^*^
	^t^p	0.228	0.072	0.059	0.387	
Neutrophils absolute (/cm)	Control group	4.85 ± 2.82	4.29 ± 3.19	3.22 ± 1.37	3.0 ± 1.30	^F^p = 0.008^*^
	ML group	3.86 ± 1.71	3.03 ± 1.75	2.56 ± 1.13	3.07 ± 1.44	^F^p = 0.015^*^
	^t^p	0.139	0.092	0.068	0.870	
Lymphocytes absolute (/cm)	Control group	2.44 ± 0.83	2.14 ± 0.63	2.03 ± 0.59	1.96 ± 0.62	^F^p = 0.007^*^
	ML group	2.45 ± 0.74	2.01 ± 0.88	1.88 ± 0.69	1.68 ± 0.53	^F^p < 0.001^*^
	^t^p	0.950	0.541	0.427	0.094	
Liver Function Tests						
ALT (IU/l)	Control group	20 (15.8–31)	23 (19–31)	22 (18–25)	23.7 (17.9–30)	^Fr^p = 0.352
	ML group	16 (13–18.2)	21.7 (17.9–27)	20 (17.5–26.3)	22 (20–31.4)	^Fr^p = 0.009^*^
	^U^p	0.051	0.356	0.473	0.992	
AST(IU/l)	Control group	24.4 (19–29)	25.7(23.4–30)	27.3(25.4–29.3)	29.8 (25.3–33)	^Fr^p = 0.161
	ML group	22 (19–24)	28.3(23–30.8)	23.3(20.9–27.3)	30 (24.5–34.7)	^Fr^p < 0.001^*^
	^U^p	0.130	0.676	0.005^*^	0.938	
Kidney Function Tests						
Serum Creatinine (mg/dl)	Control group	0.80 (0.70–1.0)	0.80(0.70–1.10)	0.80(0.70–0.90)	0.90 (0.70–1.0)	^Fr^p = 0.983
	ML group	0.70 (0.70–1.0)	0.80 (0.70–1.0)	0.90 (0.70–1.0)	0.70(0.70–0.90)	^Fr^p = 0.167
	^U^p	0.785	0.690	0.330	0.107	
Urea (mg/dl)	Control group	28(22.4–35.4)	27.9(20.1–34.6)	23.3(18.3–31.4)	22.8(19.1–33.7)	^Fr^p = 0.068
	ML group	25.2(23.3–29.1)	24.2(19.2–30.3)	23.8(21.2–31.4)	22.1(17.4–26.6)	^Fr^p = 0.277
	^U^p	0.327	0.393	0.554	0.367	
Fasting plasma glucose (mg/dl)	Control group	118.5(100–128)	112(102–129.8)	108(102.2–130.6)	115(106–125)	^Fr^p = 0.605
	ML group	104(97–112.4)	107(99.6–114.9)	103.3(96–115)	103(95–116)	^Fr^p = 0.138
	^U^p	0.067	0.426	0.165	0.123	

*RBCs* Red Blood Cells, *MCV* Mean Corpuscular Volume, *MCH* Mean Corpuscular Hemoglobin, *MCHC* Mean Corpuscular Hemoglobin Concentration, *RDW/CV* Red Cell Distribution Width—Coefficient of Variation, *PLT* Platelets, *WBCs* White Blood Cells, *ALT* Alanine Aminotransferase, *AST* Aspartate Aminotransferase. Qualitative data were described using Number and Percent, and normally, quantitative data were expressed in Mean ± SD. While non-normally distributed data was expressed in Median (IQR), Quantitative data was expressed in Mean ± SD. *SD* Standard deviation. *t* Student t-test. *U* Mann–Whitney test. *Fr* Friedman test. *F* F test (ANOVA) with repeated measures. *p* p-value for comparing between the studied groups in each period. *p*_*0*_ p-value for comparing between Baseline and After treatment in each group. * Statistically significant at p ≤ 0.0

## Discussion

The underlying mechanisms of DIC are complex, involving oxidative stress, excessive production of ROS, lipid peroxidation, and inflammatory signaling cascades mediated by NF-κB [45, 46]. Key indicators of cardiac injury associated with DOX use include biomarkers like NT-proBNP, a measure of cardiac stress and NF-κB, a regulator of pro-inflammatory mediators [47]. ML, a CysLTRA primarily used in asthma and allergic rhinitis, has emerged as a potential cardioprotective agent attributable to its potent anti-inflammatory and antioxidant characteristics [48, 49]. This randomized controlled trial is the initial to establish the cardioprotective benefit of ML versus DIC in patients with BC, providing significant clinical data, despite earlier studies mostly concentrating on preclinical models.

Both NT-proBNP and NF-κB were significantly reduced by ML. Hafez and Hassanein (2022) highlighted the role of ML in lowering cardiac stress and inflammation. Using an in vivo animal model, they showed that ML may reduce DIC by modifying oxidative stress and regulating inflammatory markers. Their research showed a noteworthy reduction in NT-proBNP levels, confirming ML's potential to reduce inflammatory reactions and related cardiovascular issues [32]. Additionally, when combined with Klotho in rats, ML had a synergistic cardioprotective effect against DIC, significantly lowering cardiac and oxidative stress biomarkers through regulating NF-κB activity and lowering cytokine expression [49]. These results are consistent with our data, which showed that the ML group's NT-proBNP concentrations significantly decreased. The significant rise in NT-proBNP levels in the control group emphasizes the cardiotoxicity of DOX, which has been associated with cumulative cardiac dysfunction in studies like those conducted by Volkova & Russell on patients with BC receiving DOX-based chemotherapy [50].

The findings of this study imply that ML might potentially lower sST2 levels, a biomarker linked to inflammation, fibrosis, and cardiac remodeling. The ML group's reported decrease in sST2 levels provides preliminary evidence of its cardioprotective benefits, especially in reducing cardiac remodeling linked to DOX therapy. Increased sST2 levels have been linked to worse outcomes for individuals with heart failure as well as those receiving anthracycline-based chemotherapy [51]. Prior research, including that conducted by Baughman on patients with heart disease, highlighted the significance of sST2 in detecting cardiac strain with its reduction being linked to improved cardiac outcomes [52, 53]. Also, the ML group's decreased sST2 levels are in line with preclinical data indicating that antioxidant and anti-inflammatory treatments can mitigate cardiac stress and remodeling [54]. Furthermore, our findings are supported by a separate preclinical study conducted in mice with transverse aortic constriction, which showed that ML improves heart function and prevents cardiac fibrosis by downregulating fibrosis-related proteins [55]. Additionally, a prospective investigation of individuals with BC receiving anthracycline-based chemotherapy revealed no significant change in sST2 levels between the baseline and the final anthracycline dosage. This could corroborate our findings on the control group, where sST2 remained unchanged, but ML may be responsible for the ML group's notable decrease in sST2 levels [56]. This study's findings suggest that ML may attenuate modestly certain aspects of cardiac remodeling associated with DOX therapy.

Although the correlation between sST2 and other biomarkers, such as NT-proBNP and NF-κB did not approach statistical significance, the direction of the correlation was positive after treatment which may support the role of ML in improving cardiac functions. This lack of significant correlation between sST2 and NT-proBNP was aligned with a clinical trial that involved patients with stable chronic heart failure during 1-year follow-up after hospitalization. The study supported the role of sST2 in cardiac risk stratification, while demonstrating no correlation between sST2 and NT-proBNP [57]. Furthermore, another study on BC patients receiving anthracycline-based neoadjuvant chemotherapy revealed no correlation between NT-proBNP elevation and sST2, while supporting the potential use of sST2 in the sensitive and early identification of cardiomyopathy linked to anthracycline-based BC chemotherapy [58]. Till now, there is no robust clinical evidence demonstrating a direct or statistically significant correlation between sST2 and NF-κB in the context of either anthracycline-induced cardiotoxicity or heart failure. In clinical setting a positive significant correlation between sST2 and C-reactive protein (CRP) as inflammatory marker was demonstrated in heart failure patients with preserved ejection fraction [59]. From a pharmacological perspective, an early responder to inflammation, NF-κB is activated in a matter of minutes to hours [60], while in chronic diseases, sST2 tends to rise later and remain elevated [61]. Therefore, even if a mechanistic link exists, it may be challenging to detect direct correlations because these two biomarkers operate on different temporal scales: NF-κB peaks early during the acute phase and sST2 rises later during the chronic or remodeling phase—especially when using single-time-point sampling, assessing different disease stages, or conducting short follow-up periods. The association between sST2 and DIC, which may entail mechanisms other than inflammation and cardiac stress, may potentially account for the non-significant correlation. As a result, sST2 might not accurately represent ML's mechanism of action. Prior preclinical and clinical research revealed that a variety of variables, such as fibrosis, cytokine signaling, and mechanical strain, affected sST2 levels. These aspects might not be entirely addressed by ML's anti-inflammatory activity alone [62–66]. To elucidate the relationship between sST2 and other cardiac biomarkers, further clinical studies are required.

The strong positive correlation between NT-proBNP and NF-κB in the two groups, especially after therapy, highlights the interconnected roles of cardiac stress and inflammation in DIC. This result aligns with earlier in vivo, in vitro, and clinical studies demonstrating that inflammatory pathways and oxidative stress are key contributors to anthracycline-induced heart damage [67]​. Although there were few clinical trials that have investigated the correlation between NT-proBNP and NF-κB, Zhang L and Hao Y, succeeded in demonstrating a positive correlation between of NT-proBNP and NF-κB expression levels in patients with acute myocardial infarction [68].

The safety outcomes of this study demonstrate that ML was well-tolerated among BC patients undergoing DOX-based chemotherapy, with no significant differences in most adverse effects between the ML and control groups, consistent with prior research showing that ML is generally safe with minimal severe side effects [69]​​. ML's anti-inflammatory property as a potential mechanism for reducing pain and discomfort are supported by the decreased incidence of lymphedema in the arms in the ML group, which points to a prospective potential benefit [49]. However, ML does not significantly exacerbate or decrease these side effects when compared to the control group, as evidenced by the non-significant changes in gastrointestinal and systemic side effects, such as diarrhea, constipation, and abdominal spasm.

The hematological parameters showed notable intra-group changes over the treatment cycles, with few significant differences between the groups that suggest that the AC regimen itself influenced parameters such as hemoglobin, RBC count, and hematocrit, rather than the addition of ML. The significant within-group changes in hemoglobin and RBC levels are consistent with the known myelosuppressive effects of DOX [70]. The absence of significant between-group differences highlights that ML did not exacerbate or mitigate these effects. Similarly, the trends in MCV, MCH, and WBCs count further confirm chemotherapy-induced hematological toxicity, consistent with findings in cancer patients undergoing chemotherapy [71]. Although both groups experienced significant declines in WBC counts over time, the lack of between-group differences suggests that ML did not significantly influence bone marrow suppression.

Regarding liver function tests, including ALT and AST, both control and ML groups showed slight increase in liver enzymes across the cycles. These fluctuations were more pronounced in ML group across the cycles. However, these changes remained within the normal reference range, suggesting no clinically significant hepatotoxicity. Since the patients were receiving DOX, such minor variations were anticipated during therapy and were not considered clinically relevant. Since DOX has been linked to up to 40% of patients experiencing asymptomatic increases in liver enzymes, and because combining it with medications like cyclophosphamide seems to increase the risk of hepatotoxicity [72]. Additionally, DOX-treated Indian cancer patients in a prospective clinical trial showed markedly elevated liver enzymes during the course of chemotherapy [73]. ML may be the cause of the ML group's significant decrease in AST during cycle 3 when compared to its corresponding control. This reduction was observed at AST level, which is a cardiac and liver-specific enzyme while was not observed in ALT which is mainly a liver-specific enzyme. This may strength the probable cardioprotective effect of ML. Although, this finding warrants further investigation but is unlikely to be clinically relevant given the overall comparable liver function profiles between the groups. ML was found to be generally safe with respect to hepatic function. Mild, asymptomatic aminotransferase elevations occur in ~ 1–2% of patients, at rates comparable to placebo and clinically apparent hepatotoxicity was rare, with only isolated cases reported, often in individuals with additional risk factors [74]. On the other hand, ML demonstrated a beneficial effect on liver health, including significant improvements in liver stiffness and enzyme levels in patients with non-alcoholic fatty liver disease [75]. Also, these findings align with previous reports in children and adults using ML for respiratory conditions, showing no significant association with hepatotoxicity [76]. Thus, the safety analysis backs up ML's favorable tolerability profile in this clinical context. Nevertheless, further large-scale clinical trials ought to be conducted to investigate the effect of ML on liver enzymes and its safety profile in special populations.

Finally, while NT-proBNP and NF-κB showed consistent trends, the interpretation of sST2 remains inconclusive and should be approached as a promising marker warranting further investigations. The study also suggested the probable cardioprotective effect of ML especially in DOX-based regimen. However, several limitations should be considered. The statistical power and generalizability of our findings may have been restricted by the comparatively small sample size. In addition, the short duration of follow-up that prevented assessment of late-onset anthracycline-induced cardiotoxicity. Furthermore, the absence of imaging-based modalities, such as echocardiography, to assess cardiotoxicity. Therefore, to validate our findings and provide a more comprehensive assessment of the role of ML against DIC in BC patients, recommendations for additional large-scale clinical trials with expanded sample sizes that incorporate other cardiac evaluation parameters and imaging are strongly warranted. Longer monitoring and follow-up times are also necessary to ascertain clinical parameters, the incidence of late cardiotoxicity, and survival outcomes. Furthermore, other relevant pathways such as apoptosis, fibrosis, and mitochondrial dysfunction were not thoroughly investigated and warrant further exploration. To properly evaluate the therapeutic potential of ML in this context and to validate the preliminary and exploratory nature of our findings, it is essential to address these gaps through larger, long-term clinical trials.

## Conclusion

This research presented as preliminary significant evidence for the cardioprotective benefits of ML in breast cancer patients receiving DOX-based chemotherapy. According to the study results, ML effectively decreased NT-proBNP and NF-κB levels, indicating that it can mitigate inflammation and DIC. The significant reduction in sST2 levels may suggest a potential role in attenuating cardiac remodeling sST2. These findings also confirm the feasibility of incorporating ML as an adjunct therapy to enhance the safety of anthracycline-based regimens However, larger and longer-term studies are needed to evaluate the broader implications of ML on survival and treatment outcomes. This study lays the groundwork for future clinical trials to confirm ML's effectiveness and long-term safety in a variety of patients by highlighting it as a promising, low-cost, well-tolerated cardioprotective drug against DIC in BC patients to improve chemotherapy safety.

## Recommendations

These findings are preliminary and need validation via other further studies with larger sample sizes and more cardiac evaluation parameters and imaging modalities. Likewise, to ascertain whether the protective benefits endure over time and whether they result in better long-term cardiovascular outcomes and survival rates, future studies should incorporate longer follow-up periods.

## Study limitations

This study is limited by its small sample size, short follow-up period, and its dependance on few biomarkers without imaging support. Late-onset cardiotoxicity and additional mechanisms were not fully assessed, warranting larger, longer-term studies with broader evaluation tools. Further larger-scale, multi-center trials are essential before ML can be routinely recommended in this setting.

## Data Availability

Accessible upon a reasonable request.

## Abbreviations

*DOX*: Doxorubicin
*ML*: Montelukast
*BC*: Breast cancer
*DIC*: DOX-induced cardiotoxicity
*NF-κB*: Nuclear factor-kappa B
*NT-ProBNP*: N-terminal Pro Brain Natriuretic Peptide
*ROS*: Reactive oxygen species
*CVDs*: Cardiovascular diseases
*CysLTRA*: Cysteinyl Leukotriene Receptor Antagonist
*CysLTs*: Cysteinyl Leukotrienes
*EDTA*: Ethylenediaminetetraacetate
*sST2*: Soluble suppression of tumorigenicity 2
*MCV*: Mean corpuscular volume
*MCH*: Mean corpuscular hemoglobin
